# Exploiting the power of UPLC in separation and simultaneous determination of pholcodine, guaiacol along with three specified guaiacol impurities

**DOI:** 10.1186/s13065-023-00949-8

**Published:** 2023-04-13

**Authors:** Hager M. Mohamed, Hala E. Zaazaa, M. Abdelkawy, Mahmoud A. Tantawy

**Affiliations:** 1 Pharmaceutical Chemistry Department, Faculty of Pharmacy, Future University in Egypt, Cairo, Egypt; 2 Analytical Chemistry Department, Faculty of Pharmacy, Cairo University, Kasr el Aini Street, Cairo, 11562 Egypt; 3 Chemistry Department, Faculty of Pharmacy, October 6 University, 6 October City, Giza, Egypt

**Keywords:** Pholcodine, Guaiacol, Coughpent® syrup, Impurities, UPLC

## Abstract

Pholcodine and guaiacol are widely used together in pharmaceutical syrups for cough treatment. On the other hand, the Ultra Performance Liquid Chromatographic technique is characterized by having the power of increasing chromatographic efficiency and decreasing run time compared to the traditional High Performance Liquid Chromatographic one. In this work, this power was exploited for the simultaneous determination of pholcodine, guaiacol along with three guaiacol impurities, namely; guaiacol impurity A, guaiacol impurity B, and guaiacol impurity E. Good separation was achieved by employing Agilent Zorbax C8 column (50 × 2.1 mm) as the stationary phase, and acetonitrile: phosphate buffer pH 3.5 (40: 60, by volume) as a mobile phase. The proposed method was validated as per International Council for Harmonisation guidelines. Linear relationships, at ranges of 50–1000 µg mL^−1^ for pholcodine and 5–100 µg mL^−1^ for guaiacol and the three related impurities, were established. Finally, the proposed method was applied for pholcodine and guaiacol determination in Coughpent® syrup and compared favorably to the reported one.

**Supplementary Information:**

The online version contains supplementary material available at 10.1186/s13065-023-00949-8.

## Introduction

Pholcodine (PHL) is 7,8-didehydro-4,5α-epoxy-17-methyl-3-[2-(morpholin-4-yl)ethoxy]morphinan-6α-ol monohydrate. It is an opioid receptor agonist used as a cough suppressant. It has a moderate sedative influence with no or little analgesic features. PHL is an official drug in the British Pharmacopoeia (BP) in which a non-aqueous titration technique is described for its assay [1]. On the reviewing literature of the last decade, PHL was determined either in a single form or combined with other drugs by several methods, namely; UV spectrophotometric [2, 3], infrared spectroscopic [4], fluorimetric [5, 6], chromatographic [7–9] and potentiometric [10, 11] ones.

Guaiacol (GUA) is chemically known as 2-methoxyphenol [1]. It has disinfectant properties, and in high concentrations is usually used as an expectorant for productive cough [12]. GUA is an official drug in BP whereas four specified impurities are stated. Those impurities are pyrocatechol (GUA impurity A), phenol (GUA impurity B), veratrole (GUA impurity C) and methyl benzoate (GUA impurity E). The BP also details liquid chromatographic method for quantification of GUA and its impurity A, and a gas chromatographic one for other impurities quantification [1]. In addition, techniques, such as; chromatography [12–14] and voltammetry [15, 16], were reported for GUA determination in the last five years.

High Performance Liquid Chromatography (HPLC) is a well-known separation technique for detecting and quantifying substances in various matrices. It is widely and commonly used to determine small amounts of pharmaceutically active ingredients in biofluids and dosage forms in a sensitive and selective manner [17–20]. On the other hand, Ultra Performance Liquid Chromatography (UPLC) is a novel system with progressive technology that evolved from HPLC. It has the benefits of small compact packing material and an upgraded pumping system accompanied by utilizing shorter columns. These features force analysis time to be faster without losing the required resolution and sensitivity [21].

The combination of PHL and GUA is directed for the symptomatic treatment of annoying dry cough. This combined dosage form makes benefits from the antitussive and expectorant actions of PHL and GUA, respectively. Upon reviewing the literature, a single reported stability-indicating reversed-phase HPLC method for their synchronous determination in pharmaceutical syrup is found [22]. This method does not take into account the concurrent determination of any official impurities. Therefore, our aim was to advance a simple and fast chromatographic method for simultaneous determination of PHL, and GUA along with three specified GUA impurities (A, B & E). To achieve this goal, a more innovative UPLC technique was selected.

## Methods/experimental

### Instruments

Agilent 1290 infinity chromatographic system comprises auto sampler with maximum 20 μL injection volume, diode array detector for UV detection, and quaternary pump for pumping solvent through Agilent Zorbax Eclipse Plus C_8_ (50 mm × 2.1 mm, 1.8 μm) column. This system is operated via OpenLAB ChemStation C.01.05 software. Sonicator (3510 Branson, UK). Electronic Balance (CPA225D Sartorius, Italy). pH meter (3505 Jenway, UK).

### Materials and reagents

#### Pure standards

PHL as well as GUA standards were obtained from Global Napi Pharmaceuticals, Egypt. Their respective purities were checked and were found to be 100.58% ± 0.88 and 100.63% ± 75 according to the official methods [1]. The three GUA impurities, GUA impurity A (98.0%), GUA impurity B (99.5%) and GUA impurity E (99.0%) were bought from Sigma-Aldrich, Germany.

#### Chemicals and reagents

Methanol, acetonitrile, phosphoric acid, and potassium dihydrogen phosphate (Sigma-Aldrich, Germany). Phosphate buffer pH 3.5; prepared by dissolving 68.0 g potassium dihydrogen phosphate in 1.0 L deionized water, pH was then adjusted by phosphoric acid to 3.5 [1], and solution was filtered using 0.2 µm filter paper by the aid of a vacuum pump. Distilled water; obtained by the aid of PURELAB® Flex Pure Water System.

#### Syrup dosage form

Coughpent® Syrup; batch No. 1875001, manufactured by Global Napi Pharmaceuticals for Penta Pharma, Egypt, obtained from the local market, and labelled to contain 6.55 mg PHL and 0.988 mg GUA per 5.0 mL syrup.

#### Solutions

Standard stock solutions of PHL (10 mg mL^−1^) and GUA (1.0 mg mL^−1^) were prepared in methanol. For GUA impurities, stock solutions of 1 mg mL^−1^ were independently prepared in methanol.

### Procedures

#### Construction of the calibration curves

Aliquots equivalent to 500–10,000 µg PHL and 50–1000 µg GUA and its three impurities (A, B & E) were exactly measured. They were then transferred into five sets of 10-mL measuring flasks. Volumes were diluted to mark by methanol. 1.0 µL from each solution has been injected into a Zorbax C8 column, pumped at flow rate of 0.2 mL min^−1^ using a solvent composed of acetonitrile: phosphate buffer pH 3.5 (40: 60, by volume) and UV detection at 270 nm. Calibration plots, relating the resulting peak area to the corresponding drug concentration, were constructed, and regression equations were calculated.

#### Application to Coughpent® syrup

One mL of Coughpent® Syrup was transmitted into 10-mL flask. Volume was then completed with methanol, and solution was sonicated for 30 min. A final solution with claimed concentration of 131.00 µg PHL and 19.76 µg GUA per mL was obtained. A 1.0 µL of this solution was injected using the previously mentioned procedures.

## Results and discussion

**Fig. 1:**
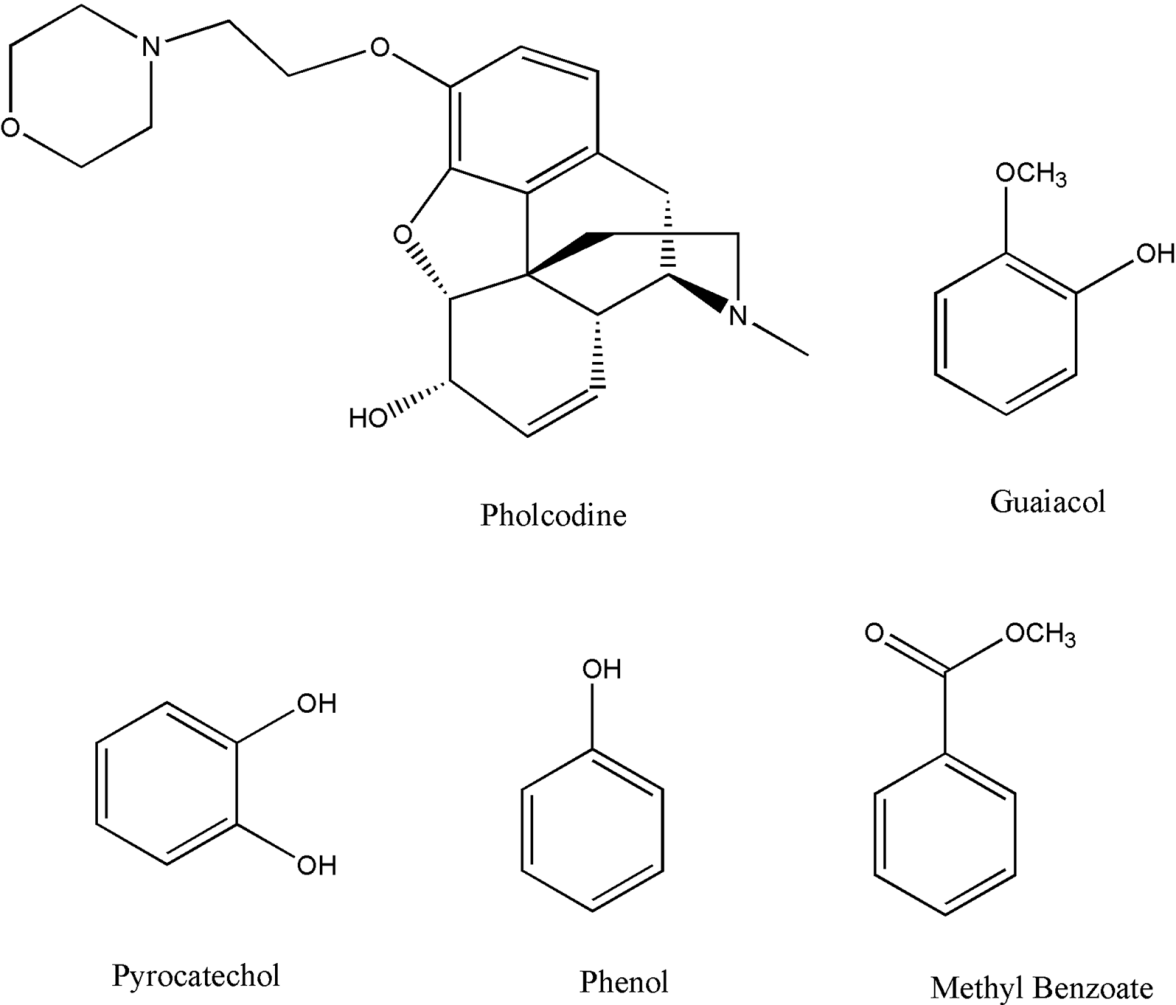
Chemical structures of the studied components

Being an advanced technology utilizing small particles in the packing of its short columns, UPLC is usually associated with high separation efficiency. In addition, solvent consumption and injection volume are smaller when compared to traditional HPLC. As a result, this technology is convenient for the pharmaceutical industry as cost reduction and fast analysis are important factors [23]. However, high instrument prices and reduced column life are considered the main disadvantages of that technology [21]. Recently, UPLC technique has been widely used in the determination of active pharmaceutical ingredients along with their impurities [24–27]. In this work, we exploited the power of this technology to develop a simple, rapid, accurate and precise chromatographic method for concurrent assay of PHL, GUA along with three specified GUA impurities. The five considered components’ chemical structures are shown in Fig. 1.

### Method development and optimization

**Fig. 2:**
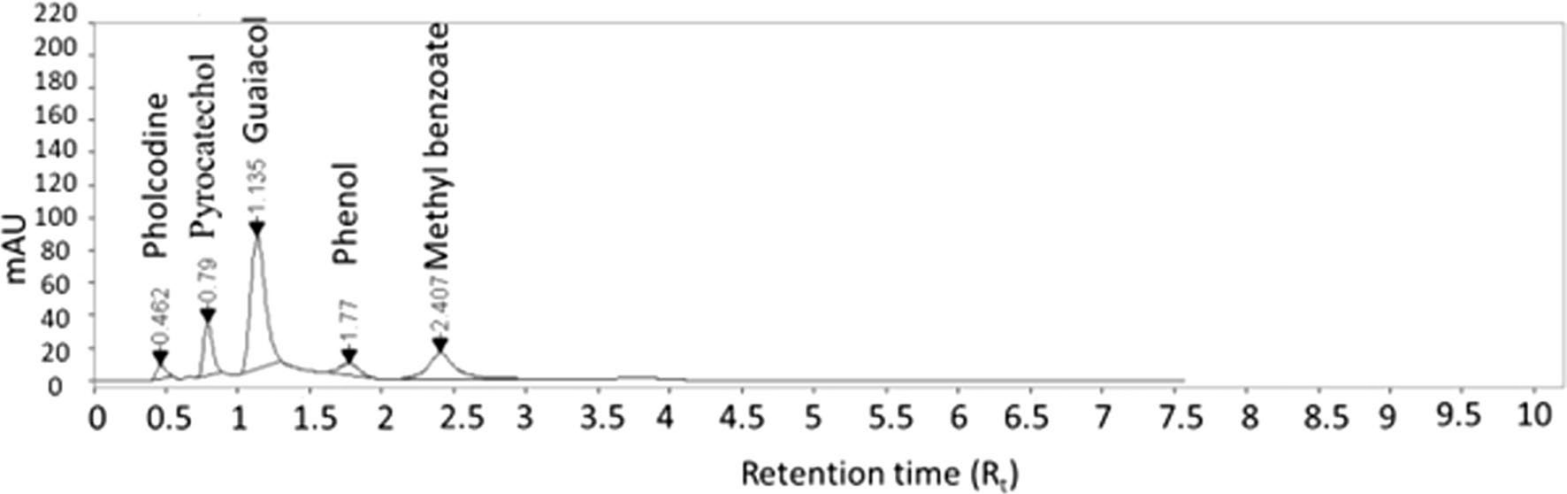
UPLC chromatogram of a resolved mixture of the studied components

Numerous trials were conducted to optimize the reversed phase chromatographic conditions. Effects of different factors, such as the pH of the used buffer, the ratio of organic solvent as well as flow rate, were studied. Upon using acetonitrile with phosphate buffers, of pH values from 4.5–7.5, in different ratios (40: 60, 50: 50 & 60: 40, by volume) as mobile phases, no separation was achieved. Separation was only achieved upon decreasing buffer pH to 3.5. This required buffer was obtained via dissolving 68.0 g potassium dihydrogen phosphate in 1.0 L deionized water and then pH was adjusted by phosphoric acid [1]. It is worth noting that acidic pH, at which PHL is ionized (pKa≈9.3) while GUA is in its neutral form, was preferred to facilitate their separation. Mobile phases of acetonitrile: phosphate buffer pH 3.5 were tried in different ratios. Unfortunately, the splitting of GUA peak was noticed upon using a ratio of 60: 40, by volume. On the other hand, broadening of PHL peak was observed upon changing the ratio to 50: 50, by volume. Peaks tailing was also encountered upon decreasing acetonitrile % to 30%. The optimized mobile phase ratio was 40 acetonitrile: 60 phosphate buffer pH 3.5, by volume. This optimized mobile phase was pumped into two types of reversed-phase columns; C_8_ and C_18_. Agilent Zorbax Eclipse Plus C_8_ column (50 mm × 2.1 mm, 1.8 μm) was found superior over C_18_ one regarding the time of analysis. These lower retention times observed are attributed to the low hydrophobicity of the C_8_ column compared to C_18_ one. For flow rate optimization, 0.1 mL min^−1^ flow rate was first applied, but broad peaks were obtained. Peaks became sharper when the flow rate was raised to 0.2 mL min^−1^. Finally, the five studied components were well resolved in less than 3.0 min using C8 column and a mixture of acetonitrile: phosphate buffer pH 3.5 (40: 60, by volume) as stationary and mobile phases, respectively. The flow rate was adjusted to 0.2 mL min^−1^ and UV detection at 270 nm. The obtained t_R_ values were 0.46, 0.79, 1.14, 1.77 and 2.41 min for PHL, GUA impurity A, GUA, GUA impurity B and GUA impurity E, respectively, Fig. 2.

### System suitability parameters

System suitability tests were conducted to verify the system’s accuracy and precision. Parameters like resolution, column efficiency, tailing, selectivity, and capacity factors were calculated. The obtained results suggested the acceptability of the method, Table 1.

**Table 1:** Parameters required for system suitability tests of UPLC method

Parameter	PHL	GUA impurity A	GUA	GUA impurity B	GUA impurity E
Retention time (*t*_R_) [min]	0.46	0.79	1.14	1.77	2.41
Resolution (*R*_s_)	NA	2.41	2.25	2.41	2.65
Tailing factor (T)	1.37	1.54	1.42	0.87	1.13
Selectivity factor (α)	NA	2.46	2.32	1.86	1.65
Column efficiency (N)	1586.15	2037.88	1657.65	1863.71	2966.25
Height equivalent to theoretical plate (HETP) [mm]	0.09	0.02	0.03	0.03	0.02

### Method validation

International Council for Harmonisation (ICH) guidelines were followed in order to validate the proposed UPLC method [28]. Regression equations parameters as well as concentration ranges for the five studied components are presented in Table 2. The table shows also LODs for the three specified GUA impurities. As shown, they are about 1.5% of the maximum GUA concertation. Satisfactory results were obtained for the analysis of pure samples, which assured the accuracy of the method. It is worth noting that the average recovery values obtained were 100.90%, 100.57%, 99.76%, 99.39%, and 99.79%, for PHL, GUA, GUA impurity A, GUA impurity B, and GUA impurity E, respectively. Precision was assessed at two levels, repeatability and intermediate precision, where three different concentrations of PHL (100, 300 and 800 µg mL^−1^), GUA and its impurities (10, 30 and 80 µg mL^−1^) were analyzed intra- and inter-daily. As shown in Table 2, low RSD % values were obtained. Moreover, mixtures comprising different proportions of the five considered components were examined to assess the specificity of the method. For robustness evaluation, some experimental factors were deliberately changed. These factors were pH of used buffer (± 0.2), ratio of organic solvent in the applied mobile phase (± 2%), and flow rate (± 0.2). Levels of a certain factor have been changed while maintaining the other chromatographic factors at their ideal levels. Good RSD % values were obtained, Table 2.

**Table 2:** Assay parameters and validation sheet for the determination of PHL, GUA, and three GUA impurities by the proposed method

Parameter	PHL	GUA impurity A	GUA	GUA impurity B	GUA impurity E	Acceptance criteria
Range	50–1000 µg mL^−1^	5–100 µg mL^−1^				NA
Slope	0.43	3.05	6.83	1.39	1.67	
Intercept	− 1.22	− 1.71	5.27	− 0.67	− 2.40	
Standard error of the slope	0.002	0.022	0.044	0.012	0.011	
Standard error of the intercept	1.374	1.334	2.454	0.639	0.667	
Correlation coefficient (*r*)	0.9998	0.9997	0.9997	0.9997	0.9997	≈ 1
Limit of detection	NA	1.64 µg mL^‒1^	NA	1.42 µg mL^‒1^	1.61 µg mL^‒1^	NA
Limit of quantification	NA	4.96 µg mL^‒1^	NA	4.31 µg mL^‒1^	4.87 µg mL^‒1^	
Accuracy (mean % ± SD)	100.90 ± 0.95	99.76 ± 0.69	100.57 ± 0.58	99.39 ± 0.51	99.79 ± 0.84	98–102%
Repeatability (RSD %)	0.93	1.00	1.04	1.31	0.89	≤ 2%
Intermediate precision (RSD %)	1.08	1.11	1.11	1.40	0.95	≤ 2%
Specificity (mean % ± SD)	100.91 ± 0.65	99.82 ± 0.60	100.68 ± 0.88	99.52 ± 0.53	99.89 ± 0.74	98–102%
Robustness:						
Peak area RSD%	1.97	1.78	1.88	1.98	1.74	≤ 2%
Retention time RSD%	0.47	0.34	0.57	0.47	0.85	≤ 2%

### Application of the proposed UPLC method in the assay of Coughpent® syrup

PHL and GUA were determined in their combined Coughpent® syrup via the proposed UPLC method. Moreover, validity of dosage form analysis procedures was assured by application of standard addition technique. Average recoveries are presented in Additional file 1: Table S1.

### Statistical analysis

PHL and GUA samples were individually assayed by their official BP methods. Results were obtained and statistically compared with those acquired by the proposed UPLC method. The calculated t- and F- values were smaller than their theoretical ones, Additional file 1: Table S2. This suggested absence of significant difference between both methods.

### Greenness study and method comparison

A comparison of our UPLC method with the reported HPLC one [22] was conducted in terms of elution time, application, and green assessment. Analytical GREEnness Metric (AGREE) was chosen as one of the most recent evaluating tools for this assessment [29]. As shown in Table 3, the advantages of the utilized UPLC technology manifested in lower analysis time and more sustainability.

**Table 3:** Greenness study and comparison of the proposed method with the reported one

Method	Elution time (min)	Application	Greenness assessment via AGREE
Reported method [22]	7	Simultaneous determination of PHL and GUA in presence of PHL oxidative degradation product	
Proposed method	3	Simultaneous determination of the PHL, GUA, and three specified GUA impurities in their quinary mixture	

## Conclusion

A novel and simple UPLC method was developed for the simultaneous quantification of pholcodine, guaiacol along with three specified guaiacol impurities (A, B and E) in Coughpent® syrup. The advanced technology of UPLC system was exploited to reduce solvent amount as well as analysis time making this proposed method an economic alternative to be used by quality control laboratories. AGREE assessment assured the sustainability of our UPLC method compared to the reported one. Validation of the proposed method showed a wide linearity range, good accuracy, precision, specificity and robustness. Furthermore, satisfactory outcomes were acquired upon applying the proposed UPLC method for the determination of active pharmaceutical ingredients in their combined syrup. Finally, t- and F- statistical tests revealed no discernible difference between our UPLC method and the official BP one.

## Supplementary Information

**Additional file 1: Table S1.** Determination of PHL and GUA in their combined dosage form and application of standard addition technique using the proposed method. **Table S2.** Statistical comparison for the results obtained by the proposed method and the official method.

## Data Availability

The datasets used and/or analysed during the current study are available from the corresponding author on reasonable request.

## Abbreviations

BP: British pharmacopoeia
GUA: Guaiacol
HPLC: High Performance Liquid Chromatography
ICH: International Council for Harmonisation
PHL: Pholcodine
UPLC: Ultra Performance Liquid Chromatography
UV: Ultraviolet
