# Supplementary material for: Exploiting the power of UPLC in separation and simultaneous determination of pholcodine, guaiacol along with three specified guaiacol impurities
Source: BMC Chem. 2023 Apr 13;17(1):35. doi: 10.1186/s13065-023-00949-8 (PMC10099691; doi:10.1186/s13065-023-00949-8)
Supplement: Supplementary file 1 — Additional file 1: Table S1. Determination of PHL and GUA in their combined dosage form and application of standard addition technique using the proposed method. Table S2. Statistical comparison for the results obtained by the proposed method and the official method. [file 13065_2023_949_MOESM1_ESM.docx]

**Additional file 1**

**Exploiting the power of UPLC in separation and simultaneous determination of pholcodine, guaiacol along with three specified guaiacol impurities**

Hager M. Mohamed, Hala E. Zaazaa, M. Abdelkawy, Mahmoud A. Tantawy

**Table S1.** Determination of PHL and GUA in their combined dosage form and application of standard addition technique using the proposed method.

| Coughpent^®^ syrup | UPLC | | |
| --- | --- | --- | --- |
| Mean^a^ ± SD | PHL | GUA | |
|  | 99.72 ± 0.48 | 99.74 ± 1.85 | |
| Standard addition technique | Claimed taken  (µg mL^‒1^) | Added (µg mL^‒1^) | Recovery % |
| PHL | 131.00 | 60 | 101.04 |
|  |  | 130 | 101.18 |
|  |  | 260 | 99.26 |
| GUA | 19.76 | 10 | 99.88 |
|  |  | 20 | 98.05 |
|  |  | 40 | 100.92 |

^a^Average of five determinations.

**Table S2.** Statistical comparison for the results obtained by the proposed method and the official method.

| Parameter | UPLC | | Official method [1] | |
| --- | --- | --- | --- | --- |
|  | PHL | GUA | PHL | GUA |
| Mean of recoveries | 100.90 | 100.57 | 100.58 | 100.63 |
| SD | 0.95 | 0.58 | 0.88 | 0.75 |
| Variance | 0.91 | 0.34 | 0.77 | 0.56 |
| *n* | 5 | 5 | 5 | 5 |
| Student’s *t*-test (2.30)^a^ | 0.54 | 0.14 | _ | _ |
| *F*-test (6.39)^a^ | 1.18 | 1.65 | _ | _ |

^a^The values in parentheses represent the corresponding tabulated values of *t* and *F* at p = 0.05

**References**

1. British pharmacopoeia. London: The Stationary Office; 2015.
